# Stepwise on-surface dissymmetric reaction to construct binodal organometallic network

**DOI:** 10.1038/s41467-019-10522-4

**Published:** 2019-06-11

**Authors:** Jing Liu, Qiwei Chen, Kang Cai, Jie Li, Yaru Li, Xiao Yang, Yajie Zhang, Yongfeng Wang, Hao Tang, Dahui Zhao, Kai Wu

**Affiliations:** 1 BNLMS, College of Chemistry and Molecular Engineering, Peking University, Beijing, 100871 China; 2 Department of Physics, The Hong Kong University of Science and Technology, Hong Kong, 999077 China; 3 Key Laboratory for the Physics and Chemistry of Nanodevices, Department of Electronics, Peking University, Beijing, 100871 China; 4 Peking University Information Technology Institute (Tianjin Binhai), Tianjin, 300450 China; 5 Beijing Academy of Quantum Information Sciences, Beijing, 100193 China; 6 CEMES, UPR CNRS 8011, 29 Rue Jeanne Marvig, 31055 Toulouse Cedex 4, France

**Keywords:** Chemical synthesis, Nanoscale materials

## Abstract

Dissymmetric reactions, which enable differentiated functionalization of equivalent sites within one molecule, have many potential applications in synthetic chemistry and materials science, but they are very challenging to achieve. Here, the dissymmetric reaction of 1,4-dibromo-2,5-diethynylbenzene (2Br-DEB) on Ag(111) is realized by using a stepwise activation strategy, leading to an ordered two-dimensional organometallic network containing both alkynyl–silver–alkynyl and alkynyl–silver–phenyl nodes. Scanning tunneling microscopy and density functional theory calculations are employed to explore the stepwise conversion of 2Br-DEB, which starts from the H-passivation of one Br-substituted site at 300 K in accompaniment with an intermolecular reaction to form one-dimensional organometallic chains containing alkynyl–silver–alkynyl nodes. Afterwards, the other equivalent Br-substituted site undergoes metalation reaction at 320–450 K, resulting in transformation of the chains into the binodal networks. These findings exemplify the achievement of the dissymmetric reaction and its practical application for controlled fabrications of complicated yet ordered nanostructures on a surface.

## Abstract (web summary)

Dissymmetric functionalization of equivalent sites in a molecule is a synthetic challenge. Here, the authors achieve an on-surface dissymmetric reaction of a molecular precursor through stepwise activation of its identical functional groups, leading to the formation of a 2D organometallic network with two types of nodes.

## Introduction

Differentiated functionalization of equivalent reacting sites within one molecule, termed as dissymmetric reaction, is of great importance for synthetic chemistry and materials science. However, its realization by wet chemistry remains a great challenge due to the homogeneity and uncontrollability of the latter. Fortunately, on-surface chemistry has evolved in the past decade to be an efficient bottom-up approach to preparing low-dimensional nanostructures with atomic precision^1–7^ and given a new horizon towards this goal. Metallic surfaces have been playing a vital role in mediating reactions. On the one hand, their anisotropic surface lattice orientations and abundant surface sites create heterogenous reaction environments for adsorbed molecules^8–10^, and their two-dimensional (2D) confining and templating effects restrict the mobilities of surface molecules on the other^5,11,12^. Tremendous effort has been devoted to exploring on-surface reactions of various organic precursors, aiming at preparations of both covalent and organometallic nanoarchitectures^2,13–20^. Most frequently, the precursors with two or more equivalent functional groups participate in symmetric reactions where identical reactions take place for all equivalent functional groups simultaneously. Several reported studies on selective on-surface activations of one C–H bond among all equivalent ones^21,22^, however, do suggest the distinguishable reactivities of these identical reacting sites under specific conditions. Most recently, on-surface dissymmetric molecular adsorption and assembly dictated by substrate lattice mismatches have been reported^23^, closing in achievement of differentiated chemical modifications of equivalent functional groups. Nevertheless, truly achieving on-surface dissymmetric reaction has yet to be explored.

Stepwise activation of the identical functional groups inside one molecule on surface is a potential solution to controllable dissymmetric reactions. Albeit their equivalence in chemical nature, the identical functional groups may possess different activation barriers on surface due to their asymmetric adsorption geometries^23^, and such a difference may be further enhanced due to the differentiated molecular configurations after the activation of the very first functional group among its counterparts^17,24^. Furthermore, to induce the inequivalent functionalization of the successively activated groups, the availability of multiple reaction pathways is indispensable, which requires the co-existence of different reactants in the reaction system. Taking aryl halides as examples, the extensively employed precursor family for on-surface syntheses dehalogenate and react with metal adatoms to form organometallic species on metal surfaces^16,25^. In the presence of abundant surface H atoms, however, the dehalogenated sites in the molecules would be H-passivated against their metalations^26–29^.

**Fig. 1:**
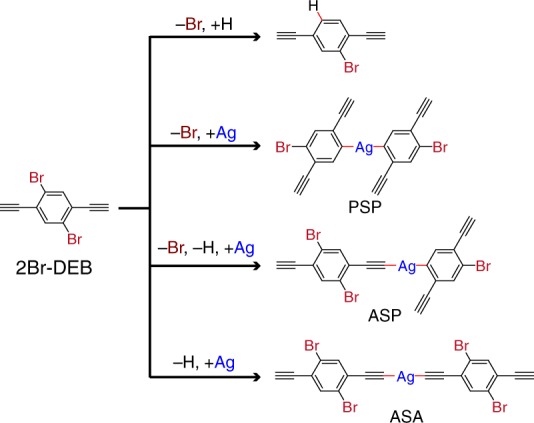
Reactions of 2Br-DEB. Possible reaction pathways and corresponding products of 2Br-DEB on Ag substrates

In this work, a stepwise activation strategy is employed to mediate the dissymmetric reaction of 1,4-dibromo-2,5-diethynylbenzene (2Br-DEB, Fig. 1) on Ag(111) which leads to a binodal organometallic network on the surface. Two terminal alkynyl groups are attached to 1,4-dibromobenzene to form the 2Br-DEB precursor, enabling to generate H atoms via their C-H bond cleavage^19,28–30^ so that both metalation and H-passivation reactions can take place at the equivalent Br-substituted sites in 2Br-DEB. In addition to the Br atoms, the terminal alkynyl groups can also undergo either homo- or hetero-intermolecular connections. Reactions of both bromides^9,31,32^ and terminal alkynes^17–19^ on Ag(111) are well documented. Possible reaction pathways for 2Br-DEB on Ag(111) are summarized in Fig. 1. Three families of organometallic products resulted from the intermolecular metalations are phenyl–silver–phenyl (PSP), alkynyl–silver–phenyl (ASP) and alkynyl–silver–alkynyl (ASA) species. Some of these reactions are involved in the conversion of 2Br-DEB on Ag(111) by sequential thermal treatment, resulting in the dissymmetric reactions of the two identical Br-substituted sites in the molecule step by step, as revealed by ultrahigh vacuum (UHV) scanning tunneling microscopy (STM) and density functional theory (DFT) calculations. The reaction forms a 2D metal-organic hybrid structure containing two types of organometallic nodes. These findings demonstrate that dissymmetric reaction can be employed to efficiently control on-surface preparations of complicated yet ordered nanostructures.

## Results

### Formation of one-dimensional (1D) ASA chains at 300 K

**Fig. 2:**
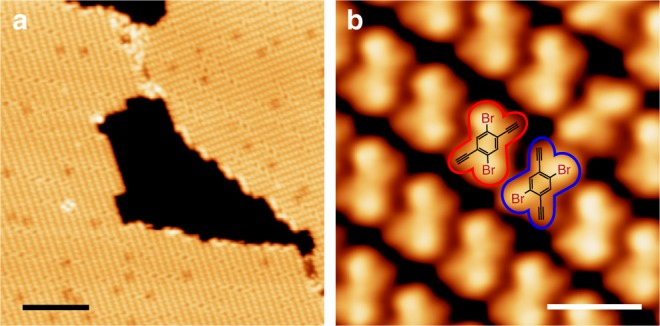
2Br-DEB monomers on Ag(111). **a** Large area (Scanning conditions: Bias = 200 mV, feedback current = 170 pA, imaging temperature = 77 K) and **b** high-resolution (−400 mV, 70pA, 77 K) STM images of the molecular assembly formed by 2Br-DEB monomers on Ag(111). Molecules of opposite adsorption chirality are marked in red and blue in **b**, respectively. Scale bars: **a** 10 nm, **b** 1 nm

Thermally deposited 2Br-DEB molecules spontaneously assembled on Ag(111) at about 150 K (Fig. 2a). An individual 2Br-DEB molecule appears as a four-lobed motif in the STM image (marked in red in Fig. 2b) where two brighter lobes are attributed to be the attached Br atoms and the other two darker ones, terminal alkynyl groups, as shown by the superimposed molecular structures in Fig. 2b. Each assembly domain is dominantly formed by molecules of the same adsorption chirality, while those of opposite adsorption chirality (marked in blue in Fig. 2b) occasionally appear as chiral defects.

**Fig. 3:**
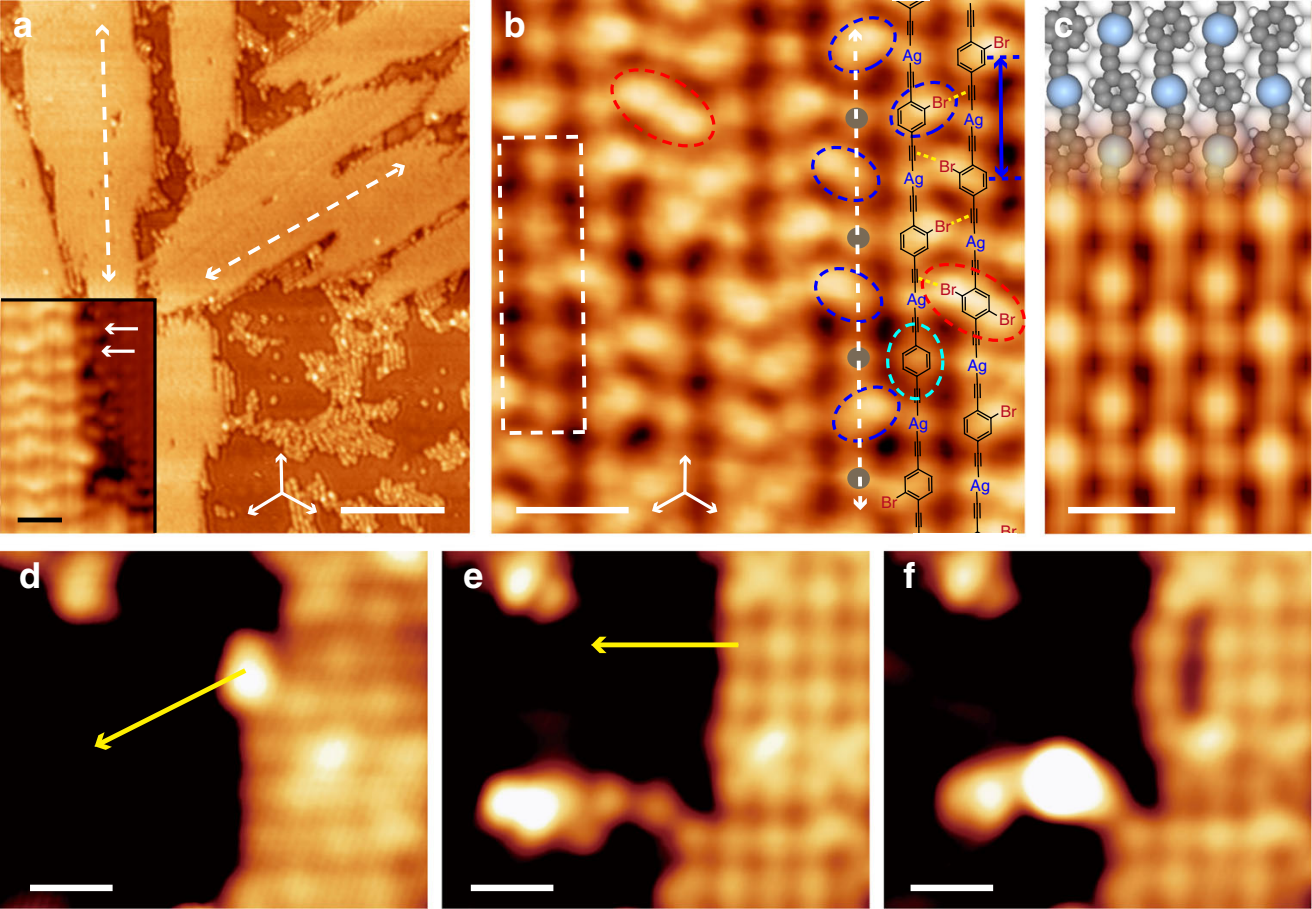
1D ASA chains formed at 300 K. **a** STM image of stripe-shaped islands formed on Ag(111) after annealing of the sample at 300 K for 8 h (200 mV, 170pA, 77 K). Inset: STM image of the periphery of a stripe-shaped island (60 mV, 180 pA, 77 K). Some detached Br atoms are representatively marked by the white arrows. **b** High-resolution STM image of the ASA chains with their structural models superimposed (60 mV, 180 pA, 77 K). Molecules with two, one and zero Br atoms attached are correspondingly highlighted with the red, blue and cyan dashed circles. Ag atoms are marked by the gray dots. The interactions between adjacent chains are marked by the yellow dashed lines. The inter-molecular distance of the ASA chain is marked by the blue arrow. The chain-segment formed by completely debrominated molecules is highlighted by the white dashed rectangle. The extending directions of the islands are marked by the white dashed arrows in **a** and **b**. The equivalent < 1$\bar 1$0 > directions of Ag(111) are marked by white arrows in **a** and **b**. **c** Optimized molecular models of the ASA chains (top) and correspondingly simulated STM image at 100 mV (bottom). Color code: gray: C, white (small): H, white (large): substrate Ag, blue: adatom Ag. **d**-**f** Sequential lateral tip manipulations of the ASA chains along the directions marked by yellow arrows (200 mV, 100pA, 4.2 K). Scale bars: **a** 20 nm, Inset: 1 nm, **b**, **c** 1 nm, **d**–**f** 2 nm

The sample was subsequently annealed at 300 K for 8 h in order to establish the reaction equilibrium. As a result, the Ag(111) substrate was covered by stripe-shaped molecular islands (Fig. 3a) which extend along equivalent < 1$\bar 1$0 > directions (white dashed arrows). High-resolution STM image (Fig. 3b) identifies alternately arranged elliptic (blue dashed circles) and circular (gray dots) protrusions along the extending direction of the stripe-shaped island (white dashed arrow). Sequential tip manipulations of the protrusions in a stripe-shaped island along the directions marked by the yellow arrows in Fig. 3d, e resulted in the bending of the molecular wires as a whole (Fig. 3d–f), indicating that the stripe-shaped islands are formed by parallelly lying molecular chains which extend along the < 1$\bar 1$0 > directions of Ag(111). Therefore, the protrusions in the chains are held together by strong chemical bonds and adjacent chains, by relatively weaker forces.

Based on the experimental facts, a structural model for the stripe-shaped islands is proposed and superimposed in Fig. 3b. In this model, the parallelly lying chains are the ASA products generated by the metalation reaction between the terminal alkynyl groups in 2Br-DEB and mobile Ag adatoms. Theoretical calculations (see Supplementary Methods for details) yield a bonding energy of −2.52 eV for the C–Ag bond in the chain, which is strong enough to survive the tip manipulations (Fig. 3d–f)^29,32^. The elliptic protrusions are due to the molecular moieties and the circular ones, Ag atoms. Three types of elliptic protrusions in different sizes can be distinguishable, as marked by red, blue and cyan dashed circles in Fig. 3b. These protrusions are assigned as the molecular moieties with two, one and zero Br atoms attached, and correspondingly denoted as 2Br-DEB, Br-DEB and DEB. The appearances of Br-DEB and DEB suggest concurrent molecular debromination and the ASA formation. The detached Br atoms appear as dim dots at the island periphery, as marked by the white arrows in the inset of Fig. 3a. The proposed model is further supported by the agreement between the measured distance between two adjacent elliptic protrusions in a chain (1.20±0.05 nm, as marked by the blue arrow in Fig. 3b) and the optimized inter-molecular distance in an ASA dimer formed by 2Br-DEB molecules (1.22 nm, Supplementary Fig. 1a). Moreover, the DFT-based STM simulation of the parallelly lying ASA chains formed by the DEB molecules is shown in Fig. 3c, depicting the same features as the experimentally observed chain-segments formed by completely debrominated molecules, as highlighted by the white dashed rectangle in Fig. 3b. Therefore, the stripe-shaped islands achieved at 300 K are assemblies of the parallelly lying ASA chains formed by partially debrominated 2Br-DEB molecules and Ag atoms. The formation of the ASA species by the reactions of the precursors possessing both halogen atoms and terminal alkynyl groups on Ag(111) under similar experimental conditions have also been reported in previous works^27–29,33,34^. The driving force for the stable parallel assembly of the ASA chains is supposed to be the attractive interactions between the electron-rich alkynyl π-bonds and the σ-hole of the Br-substituent which possesses positive electrostatic potential^35,36^, as marked by the yellow dashed lines between the ASA chains in Fig. 3b.

**Fig. 4:**
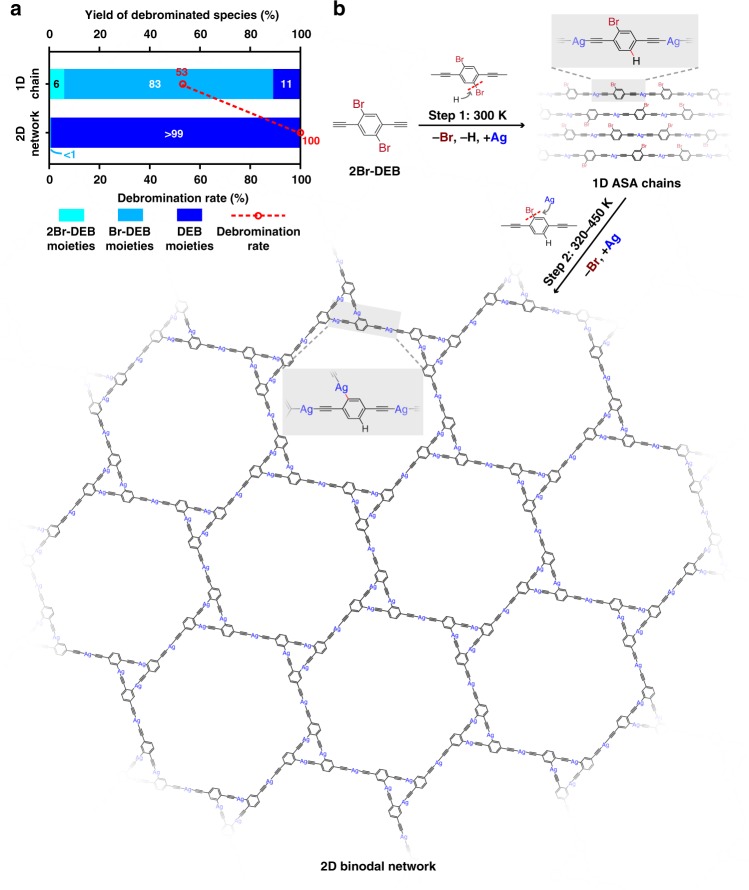
Stepwise activation of Br-substituents and dissymmetric reaction of 2Br-DEB. **a** Statistical results of the debromination yield and debromination rate in the 1D chains and 2D networks. **b** Schematic illustration of stepwise activation and dissymmetric reaction of 2Br-DEB on Ag(111)

Statistical analysis revealed that the ASA structure was the dominant reaction product at 300 K, while the ASP or PSP species were barely observed. Nearly 100% of the terminal alkynyl groups in the molecules were dehydrogenated and bonded to the Ag atoms at this stage. In addition to the intermolecular reaction between the terminal alkynyl groups, intramolecular debromination also took place at 300 K. However, in contrast to the complete conversion of the terminal alkynyl groups, about 53% Br atoms in the 2Br-DEB molecules were detached at the reaction equilibrium (Fig. 4a). The dominant debromination product was Br-DEB moieties with a yield of about 83%, while 2Br-DEB and DEB moieties occupied 6 and 11%, respectively (Fig. 4a). The relatively weak chain–chain and chain–substrate interactions, as confirmed by the above-mentioned tip-manipulations of the ASA chains, suggest that the debromination sites in the molecular moieties be passivated by H atoms^26,27^. DFT calculations also exclude the possibility of the unpassivated chains, according to the significant difference in the simulated STM image of the unpassivated chains (Supplementary Fig. 2) and their experimental counterpart. The H atoms are proposed to be originated from either the residual gas in vacuum or the dehydrogenation of the terminal alkynyl groups. Therefore, the 2Br-DEB molecules reacted on Ag(111) at 300 K to form the ASA chains mainly composed of the Br-DEB moieties. Two terminal alkynyl groups in the 2Br-DEB molecule were involved in the formation of the ASA nodes, and one Br atom was detached and the debromination site coupled with an H atom. The first step reaction is termed as Step 1 in Fig. 4b.

### Formation of 2D binodal organometallic network at elevated temperatures

**Fig. 5:**
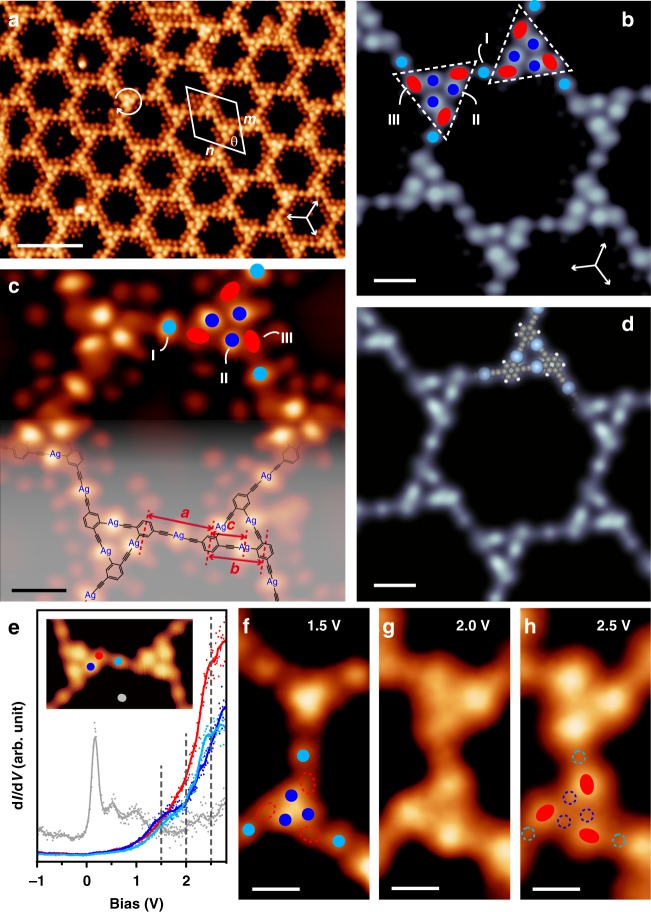
2D binodal organometallic network formed at elevated temperatures. **a** STM image of the 2D network formed by 2Br-DEB on Ag(111) after annealing of the sample at 330 K (80 mV, 190pA, 4.2 K). The unit cell is marked by the white parallelogram. The chirality of the triangular cluster is marked by the curved arrow. **b** High-resolution STM image of the 2D network (640 mV, 30pA, 4.2 K). The equivalent < 1$\bar 1$0 > directions of Ag(111) are marked by white arrows in **a** and **b**. **c** Constant-height STM image of the 2D network obtained by a Br-modified tip (500 mV, 100pA, 4.2 K). The molecular structure of the 2D network is superimposed. The distances between different protrusions are highlighted by the red arrows and marked with *a*, *b* and *c*, respectively. **d** Simulated STM image at 500 mV of the 2D network according to the optimized models as superimposed. **e** d*I*/d*V* spectra acquired on a Ag atom in an ASA node (light blue), a Ag atom in an ASP node (dark blue), a molecular moiety (red) and the substrate in a hexagonal pore (gray). Original data and smoothed results are presented by the dots and the lines, respectively. Constant-height d*I*/d*V* mappings acquired at biases of **f** 1.5 V, **g** 2.0 V and **h** 2.5 V. Scale bars: **a** 5 nm, **b**–**d**, **f**–**h** 1 nm

Further thermal treatment of the sample at 320–450 K gave rise to a remarkable transformation of the densely packed 1D phase into ordered 2D networks with hexagonal pores (Fig. 5a and Supplementary Fig. 3). The unit cell parameters of the 2D structure were measured as *m* = *n* = 3.89 ± 0.03 nm and *θ* = 60±1°. High-resolution STM image (Fig. 5b) identifies a complex porous network. Each hexagonal pore consists of six triangular clusters (white dashed triangles) and six circular protrusions (light blue dots, denoted as type I) located in between two adjacent triangular clusters. Inside the triangular clusters, there appear six protrusions, of which the inner three (dark blue dots, denoted as type II) are circular and relatively brighter, and the outer three (red dots, denoted as type III) are elliptic and relatively darker.

To uncover the delicate 2D network formed by 2Br-DEB, constant-height imaging with a Br-modified tip was conducted. The resulted STM image displayed in Fig. 5c shows that the protrusions of types I and II (light and dark blue dots) appear in similar brightness and are much brighter than the ones of type III (red dots), indicative of their different chemical nature. The dramatic difference in contrast of the three types of protrusions suggests that the 2D network be a bi-component structure. Given that the on-surface reaction solely involves the molecules and the silver substrate, one can conclude that the bi-component network is a metal-organic hybrid structure consisting of the molecular moieties and Ag adatoms. The protrusions of types I and II are identified as the Ag atoms due to their smaller size and higher brightness^31^ and the type III protrusions, the molecular moieties. To figure out how the molecules and Ag atoms are bonded in the network, spacings between the molecular moieties in the network (red arrows *a* and *b* in Fig. 5c) were measured to be *a* = 1.23±0.04 nm and *b* = 0.98±0.05 nm. These values are in good agreement with the optimized inter-molecular distances of the ASA (1.22 nm, Supplementary Fig. 1a) and ASP (0.97 nm, Supplementary Fig. 1b) dimers, supporting the judgment that the ASA and ASP nodes are formed by the molecular moieties and Ag atoms. This conclusion also finds support in the locations of the Ag atoms in between the molecules: The Ag atom between the molecules separated by a spacing of *a* sits in the right middle of two neighboring molecules, which agrees with the situation in an ASA node. The Ag atom between the molecules at a spacing of *b* locates far from one of the molecules at a distance of *c* = 0.61±0.03 nm (marked by the red arrow in Fig. 5c) which agrees well with the optimized separation between the molecule center and the Ag atom bonded via an alkynyl group in an ASP dimer (0.61 nm, Supplementary Fig. 1b). Accordingly, a molecular model with an organometallic backbone of the 2D network is proposed, as shown in Fig. 5c. In this model, two types of organometallic nodes are embedded in the network: the triangular cluster is formed by the ASP nodes between three molecules, and the adjacent triangular clusters are linked by the ASA nodes. DFT-based STM simulation of the hexagonal network at various biases, as shown in Fig. 5d and Supplementary Fig. 4b based on the structural model in Fig. 5c, is in excellent agreement with the experimental observations in Fig. 5b and Supplementary Fig. 4a. Such comparisons between the experimental and theoretical results further confirm the proposed structure for the 2D network. Other structural models with different chemical linking strategies between the 2Br-DEB molecules, including organometallic and covalent connections, were also taken into account (Supplementary Fig. 5). However, none of them agrees in dimensions with the experimental measurements (see Supplementary Discussion for details). The dim dots randomly distributed around the backbone of the network as seen in Fig. 5a–c are identified as detached Br atoms because they are prone to be moved by the STM tip (Supplementary Fig. 6).

To find further experimental support for the proposed model of the 2D network, tunneling spectra (d*I*/d*V*) experiments were performed. As depicted in Fig. 5e, the acquired d*I*/d*V* spectra on the Ag atoms (light and dark blue curves in Fig. 5e) and molecular moieties (red curve) show different characteristics: The formers exhibit small shoulder features at around 1.5 V, while the latter displays no feature at 1.5 V and a higher intensity of d*I*/d*V* above 2.0 V is instead noticed. The constant-height d*I*/d*V* mappings obtained at different biases (Fig. 5f–h) intuitively show the difference in the d*I*/d*V* spectra for the molecular moieties and Ag atoms. At 1.5 V (Fig. 5f), the Ag atoms (dark and light blue dots) are resolved more clearly than the molecular moieties (dashed red circles). On the contrary, the d*I*/d*V* mapping at 2.5 V (Fig. 5h) shows that the molecular moieties (red dots) are clearly visible but the Ag atoms (dashed light and dark blue circles) are hardly seen. The d*I*/d*V* mapping at 2.0 V (Fig. 5g) shows intermediate features of the mappings at 1.5 V and 2.5 V. As a result, the d*I*/d*V* measurements provide further evidence that the 2D network is a metal-organic hybrid structure, and reveal different locations of the Ag atoms and molecular moieties in the 2D network.

It is noteworthy that the moieties connected by the ASP nodes consisting of the triangular clusters arrange in a chiral manner, as marked by the curved arrows in Fig. 5a and Supplementary Fig. 7a. The ordered arrangement of the triangular clusters of the same chirality gives rise to chiral domains of the 2D networks. Iso-energetically, domains of the 2D networks with an opposite chirality should also appear, which was indeed experimentally observed (Supplementary Fig. 7).

Statistically, over 99% of the molecules in the 2D network have lost both Br atoms, leading to a debromination rate of nearly 100% (Fig. 4a). This means the activation of the second Br-substituents in the molecules during the second step. Meanwhile, the yields of the intermolecular reaction products, *i.e*., the ASA, ASP and PSP nodes, in the 2D structure were correspondingly about 34%, 65 and 1%. These values are similar to the ideal ones calculated from the perfect model of the 2D network, *i.e*., 33% for ASA, 67% for ASP and none for PSP. The slight discrepancy between experiment and model stems from the defects in co-existence with the ordered hexagonal networks (Supplementary Fig. 8). The significant increase in the yield of the ASP node in the 2D structure compared with that in the 1D phase indicates the involvement of the Br-substituted sites activated in the second step in the intermolecular metalation reactions. As a consequence, about 89% molecular species in the 2D network are the molecular moieties whose terminal alkynyl groups and one of its Br-substituted sites are involved in the intermolecular organometallic bonding.

**Fig. 6:**
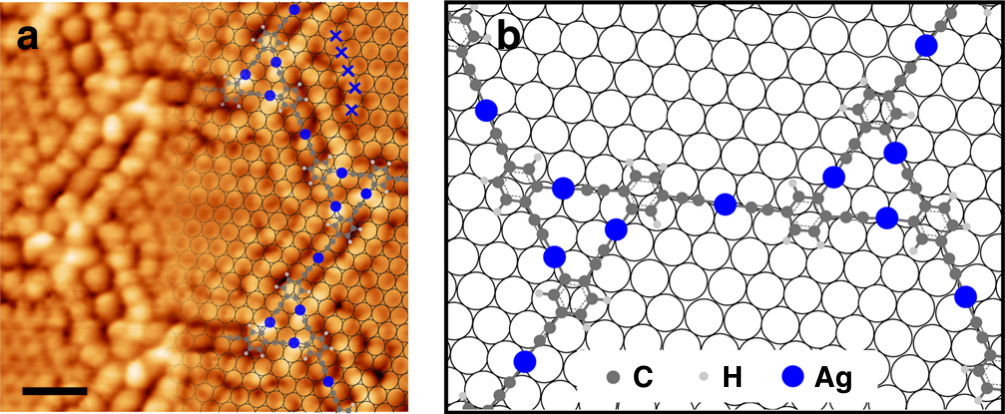
Matching relationship between the 2D network and the Ag(111) substrate. **a** High-resolution STM image of the 2D network acquired with a modified tip (380 mV, 110pA, 77 K). Molecular models and substrate lattice are superimposed. Some detached Br atoms are highlighted by the blue crosses. Scale bar: 1 nm. **b** Molecular models of the 2D network on the substrate

The transformation from the 1D phase to the 2D structure involves the dissociation of the C–Ag bonds in the ASA nodes that form the 1D chains, the diffusive rearrangement of the resulted molecular moieties, and the reformation of the organometallic bonds in the ASP and ASA nodes. The driving force for these processes lies in the metalation of the Br-substituted sites in the 2Br-DEB molecules triggered at elevated temperatures. This reaction requires the involvement of a second 2Br-DEB molecule to form a stabilized intermolecular organometallic product, which can only be fulfilled by the structural transformation of the 1D phase. Upon activation, the debromination sites may participate in metalation reaction with either the terminal alkynyl groups or the debromination sites in other molecular moieties, forming the ASP or PSP nodes, respectively. However, only the ASP nodes were found in the ordered 2D final product. This highly selective formation of the ASP species could be explained by a spectacular match between the 2D network backbone and the substrate lattice, as noticed by high-resolution STM imaging with a modified tip (Fig. 6a). As shown in the STM image (Fig. 6a), the detached Br atoms (blue crosses) were located at the hollow sites of the substrate, in accordance with previously reported result that Br atoms preferentially adsorb at the hollow sites on Ag(111) at a low coverage^37^. The so-achieved Ag(111) surface lattice is overlaid on the STM image in Fig. 6a. In addition, the molecular models of the 2D nanostructure are superimposed in Fig. 6a to clarify the backbone structure of the network imaged with a modified STM tip. A comparison of the substrate lattice and the network structure immediately manifests the surface locations of the Ag atoms in the network: All Ag atoms in the ASP nodes are located at the hollow sites of the substrate and those in the ASA nodes, at bridge sites. The molecular moieties are connected by the Ag atoms located at either hollow or bridge sites. As a result, the edges of the hexagonal pores in the 2D network run approximately along the < 1$\bar 1$0 > directions of the Ag(111) substrate, as shown by the molecular models in Fig. 6b. The remarkable match between the backbone structure of the 2D network and the Ag(111) lattice invokes a strong adlayer–substrate interaction which stabilizes the 2D surface nanostructure^19,38^. Therefore, the hexagonal 2D network in which all the Br-substituted sites activated in the second step are involved in the ASP nodes survives as the only final product with an energy preference. As a comparison, the situation where all the reactive debromination sites are engaged in the PSP nodes was also considered (Supplementary Fig. 9). However, the resulted model shows a poor match with the substrate lattice (see Supplementary Discussion for details), implying less stabilization effect of the substrate to this structure, which may possibly explain its absence. The conversion from the 1D structure to the 2D network is schematically illustrated as Step 2 in Fig. 4b.

The 2D network could survive 450 K annealing (Supplementary Fig. 10a), indicative of its high thermal stability. At higher temperatures, *e.g*., about 530 K, the organometallic network decomposed and less ordered covalent structures were formed (Supplementary Fig. 10b).

## Discussion

As described above, the two equivalent Br-substituents in 2Br-DEB are sequentially activated at different temperatures and engaged in distinct reactions on Ag(111) (Fig. 4b): One is detached at 300 K and the resulted debromination site is passivated by an H atom, while the other is detached at a higher temperature (320–450 K) to form the organometallic bond with a Ag adatom. To elucidate the process and mechanism of this dissymmetric reaction, both experimental and theoretical investigations are carried out, aiming at understanding the stepwise activation of the two Br atoms and the different reactivities of the two Br-substituted sites.

**Fig. 7:**
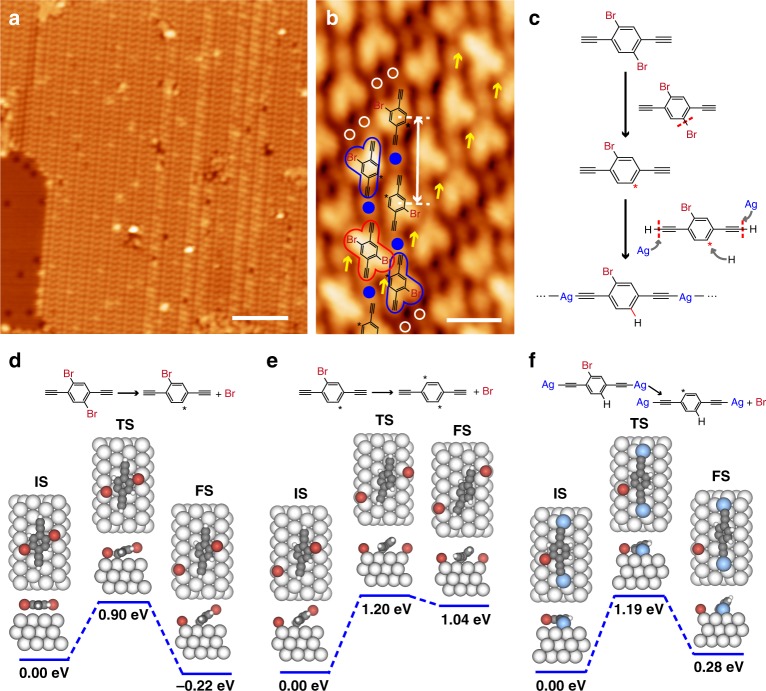
Experimental and theoretical explorations of stepwise debromination. **a** Large area (400 mV, 110pA, 77 K) and **b** high-resolution STM images (−630mV, 120pA, 77 K) of the intermediates achieved by annealing of the sample at 300 K for 40 min. Four- and three-lobed molecules are marked in red and blue in **b**, respectively. Ag adatoms and Br atoms are correspondingly highlighted by blue dots and white circles in **b**. Intermolecular distance is marked by the white arrow in **b**. Overlapped parts of the molecules are highlighted by yellow arrows in **b**. **c** Schematic illustration of the proposed reaction process of 2Br-DEB in the first step. DFT-calculated potential pathways on Ag(111) of **d** the detachment of the first Br atom in 2Br-DEB, **e** the detachment of the second Br atom in 2Br-DEB, and **f** the detachment of the Br atom in a Ag-(Br-DEB)-Ag moiety. Top and side views of the initial state (IS), transition state (TS), and final state (FS) of each reaction are depicted. Scale bars: **a** 10 nm, **b** 1 nm

Firstly, the stepwise activation of the Br atoms in 2Br-DEB on Ag(111) was studied. Experimental exploration focused on the chemical evolution of the molecule in the first reaction step at 300 K because the selective activation of one Br atom in the molecule took place at this stage. A 2Br-DEB-precovered Ag(111) sample was annealed at 300 K for only 40 min so that the molecular states at the very beginning of the first step could be scrutinized. Figure 7a displays a representative STM image of the sample thermally treated in such a way, showing a molecular structure different from either the monomer assemblies or the ASA chains. By annealing the sample at 300 K for a prolonged time, this structure vanished and the Ag(111) substrate was covered by the 1D phase, suggesting that the specific structure be an intermediate for the formation of the ASA chains. High-resolution STM image of the intermediate structure (Fig. 7b) revealed that except for a few four-lobed molecules (marked in red) that are similar in shape with intact 2Br-DEB monomers, most molecules possessed three lobes (marked in blue), implying the detachment of one Br atom. The three-lobed molecules show depressions at the debromination sites (marked by asterisks in Fig. 7b). Meanwhile, the compact arrangement of the molecules led to overlapped parts of neighboring molecules (highlighted by yellow arrows in Fig. 7b). Both facts indicate that the three-lobed molecules tilt against the surface with their debromination sites anchored to the substrate. This strong substrate-molecule interaction means that the debromination sites in the molecules are not passivated by H atoms. Therefore, the dominating product at this stage is the molecular moieties with one unpassivated debromination site with a yield of about 89%, leading to a molecular debromination rate of about 46%. These molecules co-existed with bright protrusions (blue dots in Fig. 7b) and dim spots (white circles). The former is assigned as Ag adatom and the latter, the detached Br atom. Molecules and Ag adatoms arranged alternatively in a pattern similar to that in the parallelly assembled ASA chains, but at a larger distance between adjacent molecules of about 1.6 nm (white arrow in Fig. 7b), in contrast to that in the ASA chains, 1.20±0.05 nm. Such a longer intermolecular separation suggests that the terminal alkynyl groups and Ag atoms be not chemically bonded at this early reaction stage. The co-assembly of the molecules and Ag atoms are highly likely to be stabilized by the non-bonding interaction between the terminal alkynyl groups and Ag adatoms, as reported in our previous work^39^. These results demonstrate that the organometallic reaction between the terminal alkynyl groups and Ag atoms come into play after the formation of the intermediate structure, requiring a longer annealing time at 300 K.

Based on the experimental observations, the conversion process of 2Br-DEB in Step 1 at 300 K is proposed as follows (Fig. 7c): The reaction starts from the debromination of one Br atom in the molecule, resulting in unpassivated Br-DEB moieties as an intermediate structure in the first reaction step. Following that, the metalation reaction takes place between the terminal alkynyl groups and Ag adatoms. Simultaneously, the debromination site in the molecule is passivated by an H atom before the debromination of the second Br atom. As a result, the ASA chains composed of the passivated Br-DEB molecules become the equilibrium product at 300 K.

By using the experimentally observed precursor monomer and intermediate structure as the initial and final states, respectively, DFT calculations for the detachment of the first Br atom in 2Br-DEB were carried out. The resulted potential pathway (Fig. 7d) shows an energy barrier of 0.90 eV, which is in accordance with the reaction temperature of 300K^34,40^. The resulted molecular moiety (FS) tilts against the surface with the debromination site anchored to the substrate, leading to an increase of 1.48 Å in the distance between the remaining Br atom in the molecule and the substrate, compared with that of the precursor monomer. Due to this significant lifting of the attached Br atom off the substrate, an enhanced barrier of 1.20 eV is found for the activation of the second Br atom (Fig. 7e), which corresponds to a reaction temperature much higher than 300 K^40^. Consequently, the detachment of the second Br atom in an unpassivated molecular moiety is blocked at 300 K, leading to a high yield of the unpassivated Br-DEB moieties as the intermediate species. The rigid backbone of 2Br-DEB, *i.e*., the benzene ring, plays a key role in suppression of the second debromination reaction at this stage by adopting a tilted configuration of the molecule after the detachment of the first Br atom. As a comparison, 2,5-diethynyl-1,4-bis(4-bromophenylethynyl)benzene (2Br-DEBPB), a similar precursor as 2Br-DEB used in our previous work^29^, which also possesses two alkynyl groups and two equivalent Br atoms but a flexible backbone underwent the debromination reactions simultaneously.

The above DFT calculations explain the selective activation of one Br atom in the intermediate species. Further calculations were performed to understand the suppressed detachment of the second Br atom in the molecules which consist the ASA chains with their debromination sites passivated by H atoms. First of all, the potential pathway of the debromination reaction of a passivated monomeric Br-DEB molecule was calculated (Supplementary Fig. 11). The results indicate that the reaction is exothermic with a barrier of 0.88 eV. Following that, the molecular model applied as the initial state was then replaced by a Br-DEB moiety with both terminal alknyl groups bonded to Ag adatoms in order to simulate the molecular species forming the ASA chains (Fig. 7f). The calculated potential pathway (Fig. 7f) displays a debromination barrier of the endothermic reaction of 1.19 eV. Such a barrier is too high for the reaction to take place at 300 K^40^, which explains the suppressed detachment of the second Br atom at the temperature. The significant increase in the debromination barrier of the Br-DEB moiety bonded to the Ag adatoms with respect to that of the monomeric molecule suggests that the formed ASA chains play a key role in the suppression of the detachment of the second Br atom at 300 K. This effect of the 1D chains is attributed to the restricted motions of the molecules in the chains. An efficient activation of the second Br atom in the molecules only becomes conspicuous at 320 K or higher where the molecular mobility is substantially enhanced. Similar phenomena that the reaction barriers increase due to the restricted motion of molecules on surfaces were also observed in other on-surface reactions^9^.

**Fig. 8:**
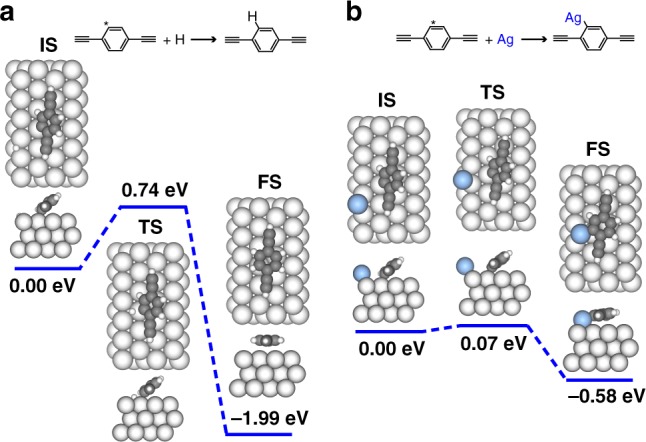
Theoretical investigations of different reactivities of the debromination sites. DFT-calculated potential pathways on Ag(111) of **a** the coupling between an unpassivated DEB molecule and an H atom, and **b** the reaction between an unpassivated DEB molecule and a Ag adatom

Secondly, we focus on the differentiated reactivities of the Br-substituted sites activated at different temperatures. The calculated potential pathways of the coupling reactions of an unpassivated DEB molecule with either an H atom (Fig. 8a) or a Ag atom (Fig. 8b) are compared. The DFT calculations point out that the coupling between an unpassivated DEB moiety and an H atom is an exothermic reaction (−1.99 eV) with an energy barrier of 0.74 eV (Fig. 8a). Although a smaller energy barrier of 0.07 eV for the reaction between the debrominated molecules and Ag adatoms also exists in theoretical calculations (Fig. 8b), the low energy barrier for the reverse process of the molecule-Ag coupling (0.65 eV) warrants the reversible reaction process at 300 K. Therefore, with abundant H atoms generated by the simultaneous dehydrogenation of the terminal alkynyl groups at 300 K and from the vacuum, the final reaction product at the temperature would be the H-passivated molecules rather than the molecules bonded with Ag adatoms. In addition, the tiny size and low diffusion barrier on Ag(111)^41^ of the H atoms facilitate their diffusion between the parallelly lying ASA chains formed at 300 K, and thus promote their approaching to the debrominated molecules embedded in the chains. As a comparison, the relatively larger size and higher diffusion barrier^42^ of the Ag adatoms impede their availability for the molecule-Ag reactions at 300 K. Consequently, most debrominated molecules are engaged in the coupling with the H atoms, and hence the molecule-Ag reaction is suppressed at 300 K. At higher temperatures, however, the molecule-Ag reaction is thermally triggered. The reasons are twofold: On the one hand, the diffusivities of both the Ag adatoms and molecular moieties are enhanced at the elevated temperatures, increasing the approaching probability of the Ag adatoms to the debrominated molecules. The Ag-molecule reaction product could be stabilized by the bonding of the Ag atom to another molecular moiety to form a strong intermolecular organometallic connection. On the other hand, the lack of the H atoms due to the complete conversion of the terminal alkynyl groups at 300 K followed by the desorption of H atoms at the high temperatures suppresses the competing molecule-H reaction. As a result, the reaction between the debrominated molecules and the Ag adatoms becomes dominating at elevated temperatures.

The competing reactions of H and Ag atoms with halides have been reported in other on-surface systems^26,28,29^, but none of them led to dissymmetric reaction product. We propose that the dissymmetric reaction observed in this work is promoted by the stepwise activation of the two Br atoms in 2Br-DEB so that different reactions of the debromination sites, *i.e*., C–H coupling and C–Ag bond formation, could take place separately at different temperatures. This proposal receives support from the fact that no dissymmetric reaction of the two equivalent Br-substituted sites can be observed in the reaction of 2Br-DEBPB on Ag(111) where the two Br atoms are indiscriminately activated, as reported in our previous work^29^.

To summarize, the dissymmetric reaction of the bi-functional 2Br-DEB precursor was achieved on Ag(111) in a stepwise manner. Combined STM measurements and DFT calculations revealed distinct reactions of the two equivalent Br-substituted sites in the molecule which were triggered at different temperatures: The one activated at 300 K coupled with an H atom, while the other underwent metalation reactions at 320–450 K. The 2Br-DEB molecules underwent chemical reactions to realize successive formations of the 1D ASA chains at 300 K and the 2D binodal organometallic network at elevated temperatures. These findings shed light on the fine-tuning methodologies of on-surface chemistry and open up another avenue to fabricate complicated yet ordered nanostructures via the dissymmetric reaction strategy by using carefully designed precursors and precisely controlled reaction processes.

## Methods

### Sample preparation and STM measurements

All STM experiments were performed with a Unisoku UHV-STM with a background vacuum better than 2 × 10^–10^ Torr. The atomically flat Ag(111) surface was prepared by repeated cycles of Ar^+^ sputtering and annealing at about 780 K. 2Br-DEB molecules (synthesized according to ref. ^43^) were evaporated at room temperature from a tantalum boat onto the clean substrate kept at about 150 K. The coverage was controlled to be lower than 0.6 ML. The sample was subsequently annealed up to different temperatures. The STM tip was an etched W wire (ϕ 0.25 mm) and was cleaned by e-beam heating in UHV. All STM images were acquired at 4.2 K or 77 K in constant current mode unless otherwise specified and processed by the WSxM software^44^. All d*I*/d*V* measurements were conducted by using a lock-in amplifier with a modulation of 15 mV (rms) and a frequency of 1.2 kHz at 4.2 K.

### Calculation methods

DFT calculations of the potential pathways were conducted with Climbing Image Nudged-Elastic Band (CI-NEB) method by using the 5.3.5 version of the Vienna Ab initio Simulations Package (VASP)^45–47^. The pseudopotentials constructed according to the projector augmented wave (PAW) method^48^ were employed to describe the core electrons. The van der Waals (vdW) dispersive correction was taken into account by calculation of the exchange-correlation energy with the opt-B88 functional^49–51^. The molecule was relaxed on a fixed Ag(111) substrate which was modeled by a slab of 72 atoms which form 3 atomic layers. A vacuum layer of 15 Å was applied between neighboring slabs to ensure their decoupling. The atom positions were relaxed until the maximum force on each atom was smaller than 0.05 eV Å^−1^. A Γ centered grid was applied for the Brillouin zone sampling.

DFT optimization of the models of the 1D ASA chains (passivated and unpassivated) and the reduced model (trimer) of the 2D network were also performed by using VASP^47^, with pseudopotentials described by the PAW method^48^. The exchange-correlation energy was calculated by generalized gradient approximation in the framework of the Perdew–Burke–Ernzerhof (PBE) functional^52,53^. The kinetic energy cutoff was set to 440 eV. The van der Waals dispersive correction was considered with the DFT-D2 method^54^ for the molecule-molecule and molecule-surface interactions. The surface was described by a four-atomic-layer slab of 64 atoms with the dimension of the supercell of 11.8 Å × 10.3 Å × 27.4 Å for the 1D chains, and by a three-atomic-layer slab of 240 Ag atoms with a 23.0 Å × 24.9 Å × 27.4 Å supercell for the trimer. For the Brillouin zone sampling, a 2 × 2 × 1 Monkhorst–Pack grid^55^ was used during the geometrical optimization for the 1D ASA models, while the Γ point was used for the trimer model. During the optimization, except for the Ag atoms in the two bottom layers of the slabs that were fixed, all other atoms were fully relaxed until the force on each of them was smaller than 20 meV Å^−1^. The electronic self-consistent field (SCF) step was converged within 0.001 meV.

After the model optimization, the STM images of the 1D ASA chains and the 2D network were simulated using GREEN, a program based on extended Hückel theory^56^. The substrate-molecule-STM tip junction was explicitly described. The optimized geometries of the 1D ASA chains were integrated directly by adopting a (4 2/0 4) surface unit cell. The geometry of the 2D network was built by duplication of the optimized trimer and integrated into a (10 5/−10 15) surface unit cell. The STM tip apex was describe by a W(111) cluster of 10 atoms. At each (*x*,*y*,*z*) position of the tip, the transmission coefficient was evaluated through a Green-function approach^57^, and the tunneling current was calculated by applying the Landauer–Büttiker formula^58^. All STM simulations were processed by the WSxM software^44^.

## Supplementary information

Supplementary Information

## Data Availability

The data that support the findings of this study are available from the corresponding authors upon reasonable request.
