# Supplementary material for: Stepwise on-surface dissymmetric reaction to construct binodal organometallic network
Source: Nat Commun. 2019 Jun 11;10:2545. doi: 10.1038/s41467-019-10522-4 (PMC6560069; doi:10.1038/s41467-019-10522-4)
Supplement: Supplementary file 1 — Supplementary Information [file 41467_2019_10522_MOESM1_ESM.pdf]

Supplementary Information for

Stepwise On-Surface Dissymmetric Reaction to Construct Binodal Organometallic Network

Liu *et al.*

SUPPLEMENTARY FIGURES

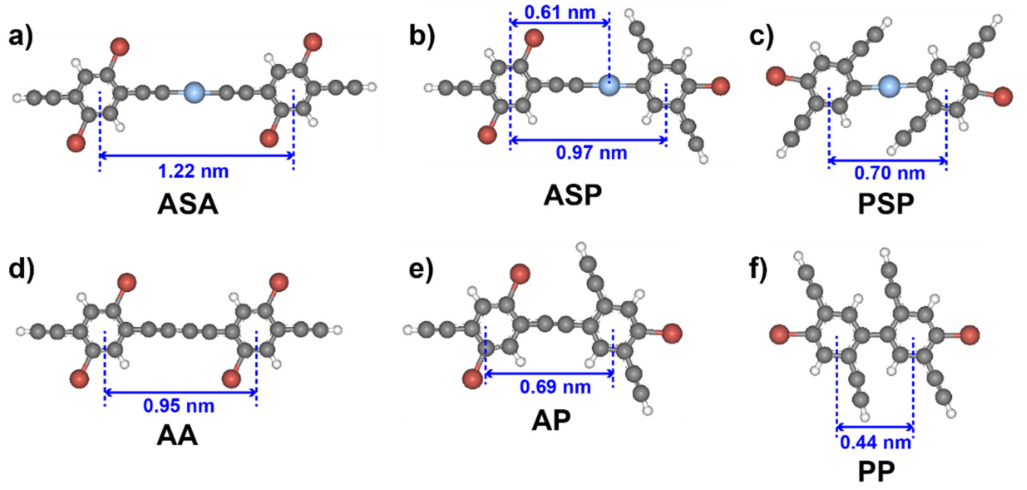

Supplementary Figure 1. Theoretically optimized models. Optimized models of (a) an ASA dimer, (b) an ASP dimer, (c) a PSP dimer, (d) a Glaser coupling (AA) dimer, (e) an Ullmann coupling (PP) dimer, and (f) a Sonogashira coupling (AP) dimer. The optimized dimensions of the structures are marked by numbers.

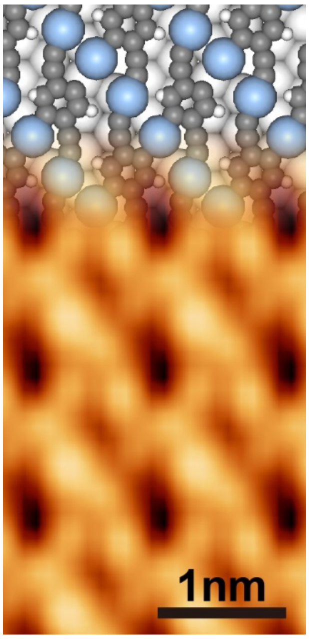

Supplementary Figure 2. DFT-calculated unpassivated ASA chains. DFT-optimized molecular models of the unpassivated ASA chains (top) and the accordingly simulated STM image at 100 mV (bottom). Color code: grey: C, white (small): H, white (large): substrate Ag, blue: adatom Ag.

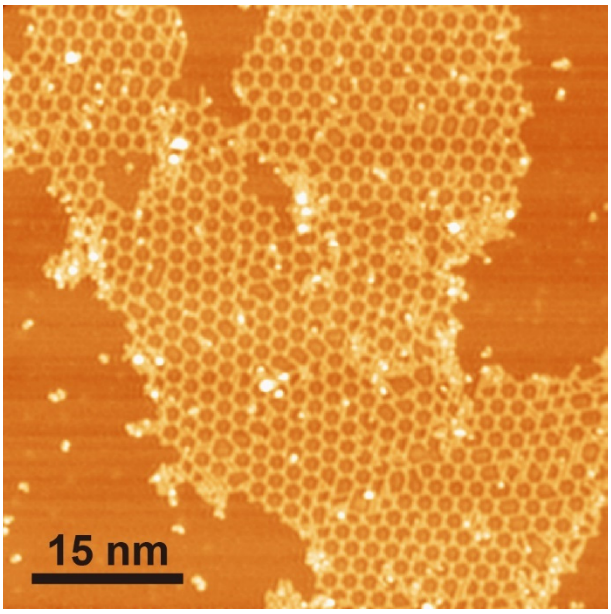

Supplementary Figure 3. 2D networks. Large area STM image of the 2D network formed by 2Br-DEB on Ag(111) after annealing of the sample at 420 K (10 mV, 80 pA, 77 K).

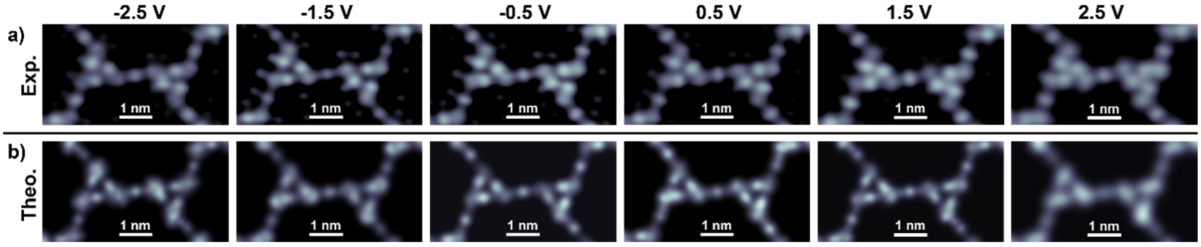

Supplementary Figure 4. Comparison between experimental and simulated STM images. (a) Experimental (100 pA, 4.2 K) and (b) simulated STM images of the 2D structure at (from left to right) -2.5 V, -1.5 V, -0.5 V, 0.5 V, 1.5 V and 2.5 V.

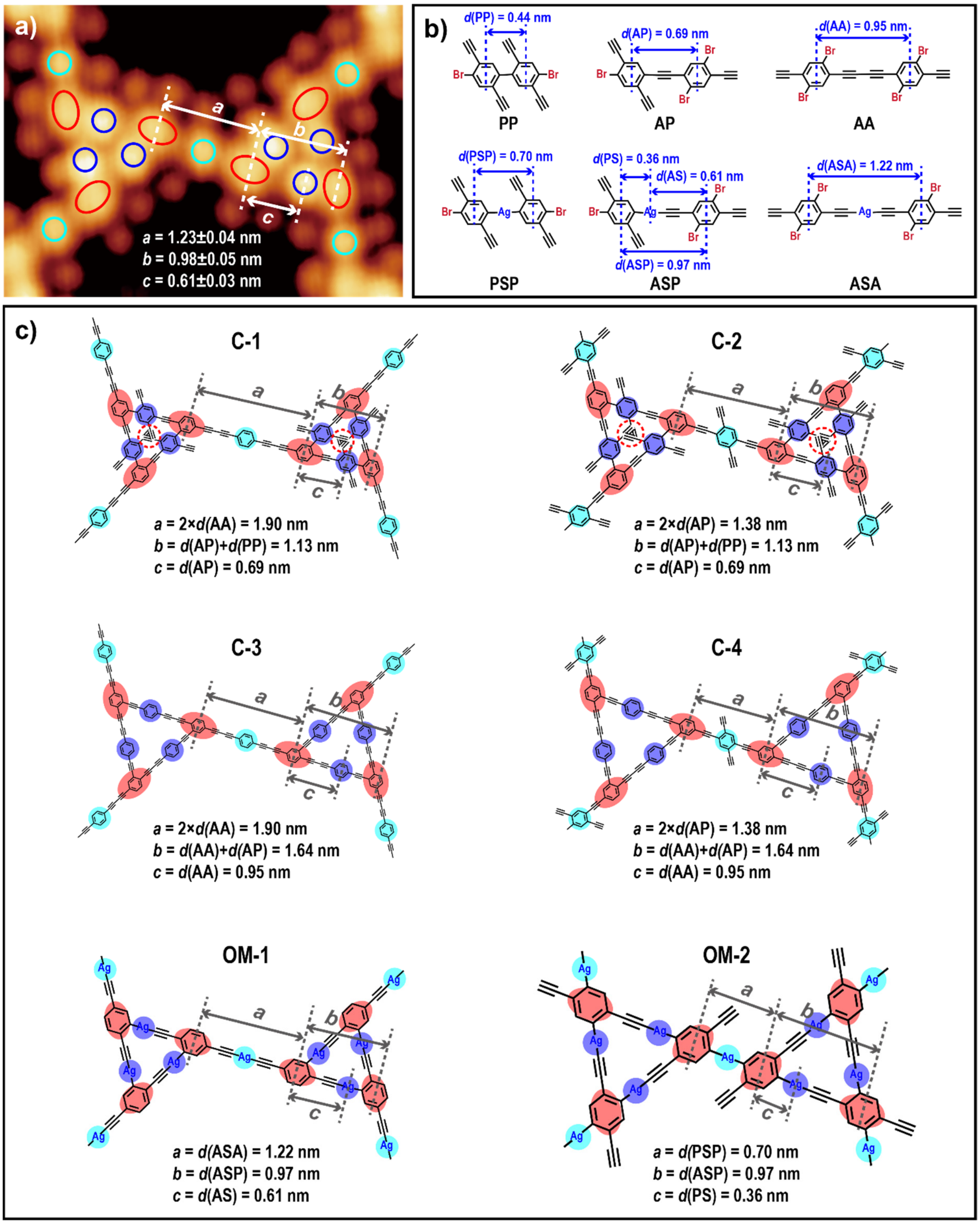

Supplementary Figure 5. Other chemical structures proposed for the 2D network. (a) High-resolution STM image of the 2D network formed by 2Br-DEB on Ag(111) (640 mV, 30 pA, 4.2 K). (b) Molecular structures of the covalent and organometallic dimers formed by 2Br-DEB with their dimensions marked in blue. (c) Proposed molecular models with similar backbone structures as that of the 2D network observed by STM.

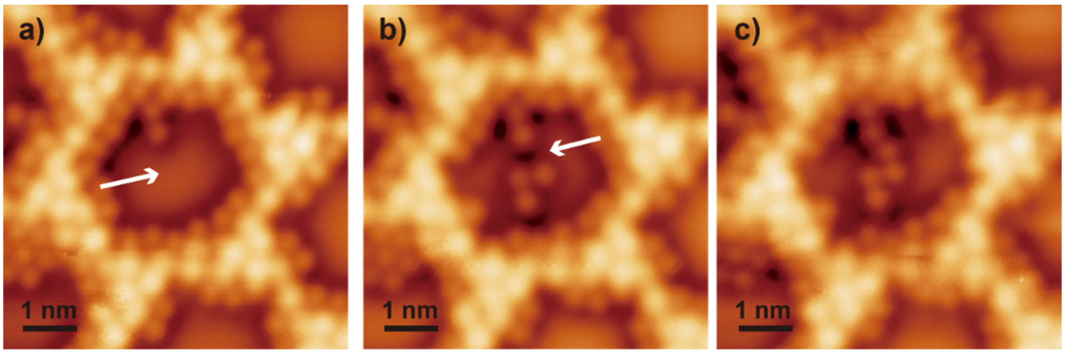

Supplementary Figure 6. Tip manipulations of detached Br atoms. (a) STM image of the 2D network with dim dots randomly distributed around the backbone (90 mV, 190 pA, 4.2 K). Lateral tip manipulation of a dim dot is performed along the direction marked by the white arrow. (b) STM image of the same area after the first manipulation (90 mV, 190 pA, 4.2 K). A second trial is then conducted along the direction marked by the white arrow. (c) STM image of the same area after the second manipulation (90 mV, 190 pA, 4.2 K).

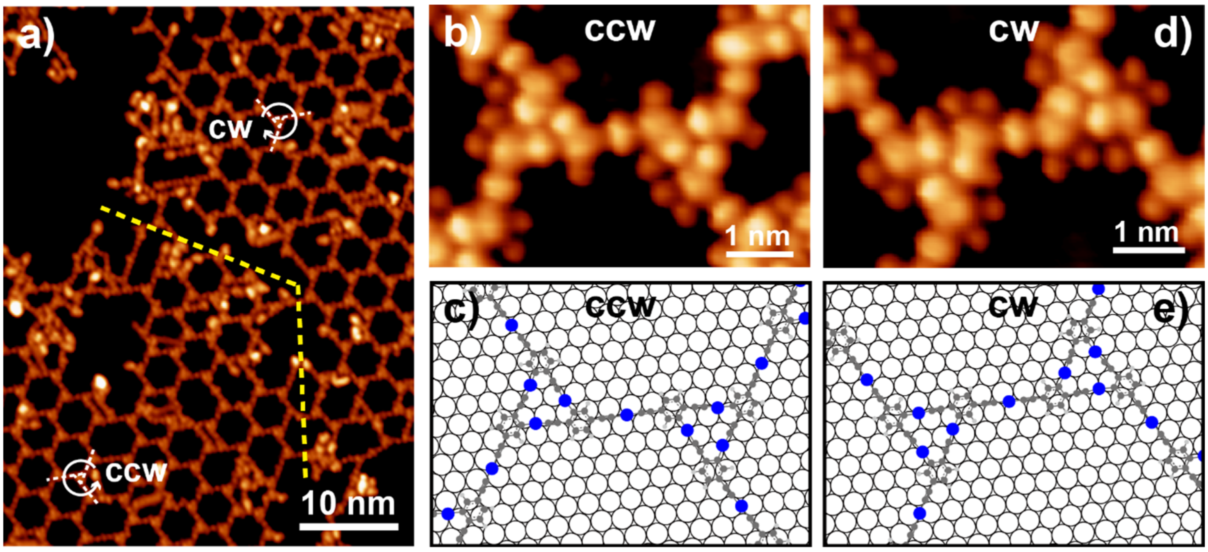

Supplementary Figure 7. Chirality of the 2D network. (a) STM image of two chiral domains of the 2D network (1600 mV, 30 pA, 4.2 K). (b) High-resolution STM image (250 mV, 80 pA, 4.2 K) and (c) molecular model of the network with the CCW triangular clusters. (d) High-resolution STM image (250 mV, 80 pA, 4.2 K) and (e) molecular model of the network with the CW triangular clusters.

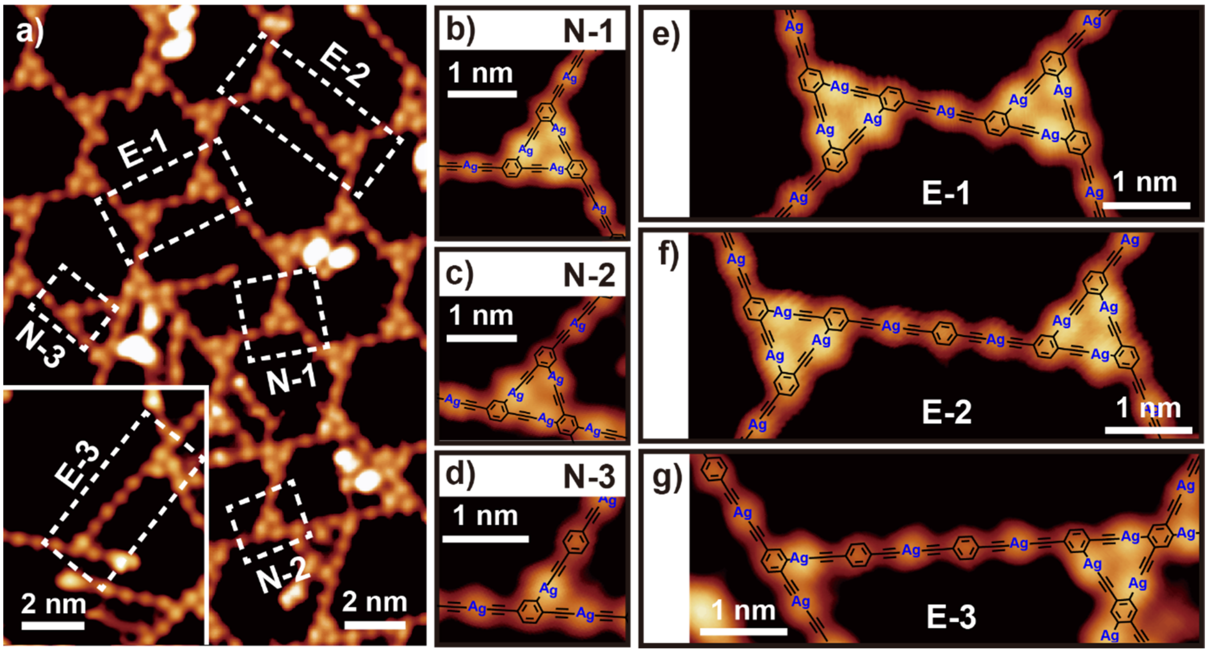

Supplementary Figure 8. Defect structures in co-existence with the ordered 2D network. (a) STM image of the defect structures in co-existence with the ordered 2D network formed by 2Br-DEB on Ag(111) (10 mV, 100 pA, 4.2 K). Inset: 10 mV, 100 pA, 4.2 K. The structural units N-1, N-2, N-3, E-1, E-2 and E-3 (inset) are highlighted by white dashed rectangles. High-resolution STM images of (b) N-1 (10 mV, 100 pA, 4.2 K), (c) N-2 (10 mV, 100 pA, 4.2 K), (d) N-3 (10 mV, 100 pA, 4.2 K), (e) E-1 (10 mV, 100 pA, 4.2 K), (f) E-2 (10 mV, 100 pA, 4.2 K), and (g) E-3 (10 mV, 100 pA, 4.2 K). The molecular structures of the structural units are superimposed in (b-g).

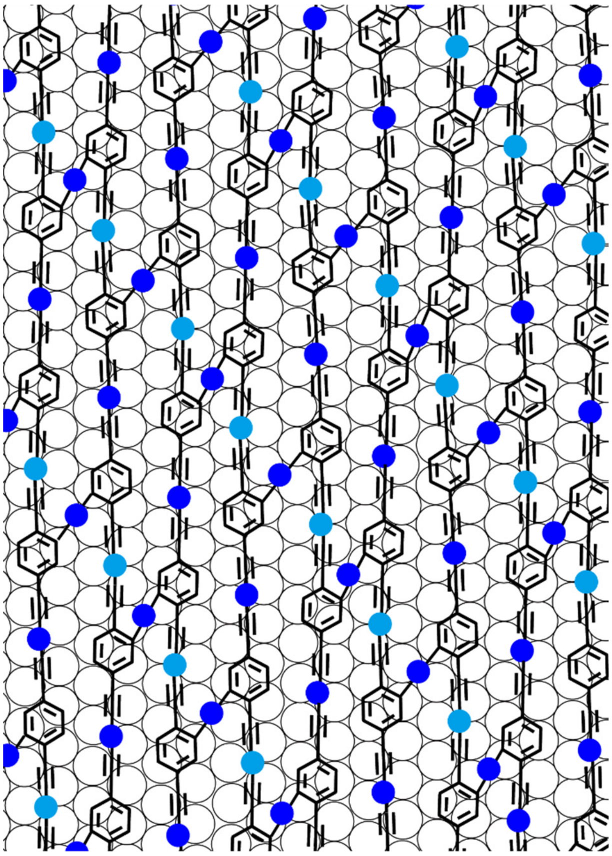

Supplementary Figure 9. Model for the 2D structure with all debromination sites involved in the PSP nodes. Proposed model of the 2D structure where all Br-substituted sites activated in the second step are involved in the PSP nodes

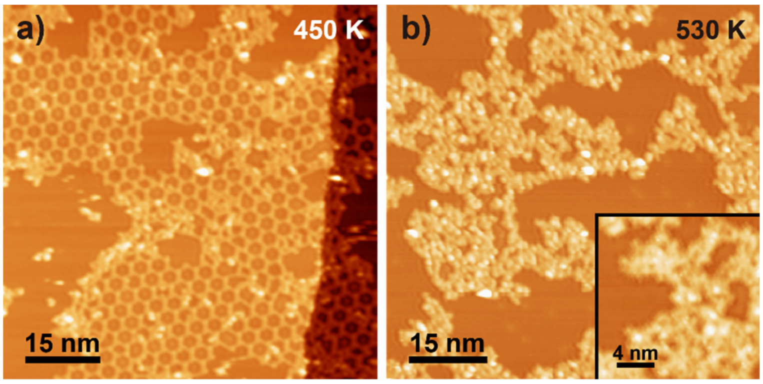

Supplementary Figure 10. Structures formed at elevated temperatures. Representative STM images of the 2Br-DEB pre-covered Ag(111) sample after annealing at (a) 450 K (100 mV, 70 pA, 77 K), and (b) 530 K (10 mV, 80 pA, 77 K), respectively.

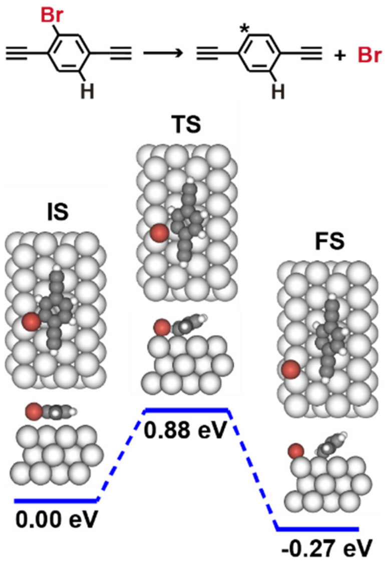

Supplementary Figure 11. Calculated pathway for the debromination of monomeric Br-DEB. DFT-calculated potential pathway for the detachment of the Br atom in monomeric Br-DEB on Ag(111).

SUPPLEMENTARY METHODS

Optimized Models of Relevant Species and the C-Ag Bonding Energy in an ASA Dimer.

The geometries of three organometallic dimers (ASA, ASP and PSP) and three covalent dimers (AA, AP and PP) formed by 2Br-DEB in gas phase were optimized by density functional theory (DFT)[1,2] calculations with the Gaussain09 software[3] using B3LYP functional[4,5]. The 6-31g(d,p) basis set was employed for C and H atoms in all species, and LANL2DZ basis set with the corresponding effective core potential (ECP)[6-9] were used for Br and Ag atoms. The optimized models of these structures are shown in Supplementary Figure 1 with their dimensions marked.

By using the same method, the geometry optimization and frequency calculations of a simplified ASA dimer formed by two alkynyl benzene radicals connected by one Ag atom were carried out. The energy of the C-Ag bond in the ASA dimer was calculated as below:

$$E_{\text{C-Ag}} = (E_{\text{ASA dimer}} - E_{\text{Ag}} - 2E_{\text{radical}})/2 \quad (1)$$

where $E_{\text{ASA dimer}}$, E_{Ag} and E_{radical} correspondingly refer to the relaxed energy of the ASA dimer, silver atom and molecular radical. As a result, $E_{\text{C-Ag}}$ = -2.52 eV. All energies were reported by the addition of an unscaled zero-point energy (ZPE) correction.

SUPPLEMENTARY DISCUSSION

Other Chemical Structures Proposed for the 2D Network.

In addition to the molecular structure of the hexagonal network shown in Fig. 5c, other models possessing similar backbone structures as that observed in STM images (Supplementary Figure 5a) are also proposed based on all possible chemical linkings between 2Br-DEB molecules, including organometallic and covalent ones (Supplementary Figure 5b).

In total, six molecular structures are proposed, including four covalent ones (C-1, C-2, C-3 and C-4 in Supplementary Figure 5c) and two organometallic ones (OM-1 and OM-2). Featured lengths marked as *a*, *b* and *c* of each structure are calculated according to the optimized dimensions of the involved structural units, as marked in Supplementary Figure 5b. Taking the length *a* of structure C-1 as an example, the intermolecular distance marked as *a* in C-1 is the distance between two molecules separated by two alkynyl-alkynyl (AA) connections. As a result, *a* is calculated to be twice as the optimized intermolecular distance of an AA dimer, *i.e.*, 0.95 nm, leading to a = 1.90 nm. The so-achieved calculated values of *a*, *b*, and *c* of the six proposed models are marked below each structure in Supplementary Figure 5c. Comparisons between the calculated featured lengths of the proposed models and the measured values of the 2D network, as marked in Supplementary Figure 5a, indicate that none of the structures except OM-1 shows featured lengths that agree with the corresponding experimental measurements. Moreover, C-1 and C-2 display huge steric hinderance inside their triangular clusters due to the overlapped molecular groups, as highlighted by the red dashed circles in C-1 and C-2 models. As for structure OM-1, that is, the same as that illustrated by the model in Fig. 5c, all featured lengths are in excellent agreement with the correspondingly measured values. In conclusion, all molecular structures proposed for the 2D network except OM-1 are excluded due to their different dimensions compared with that of the 2D structure observed by STM, leaving OM-1 the only rational structure for the 2D network.

Defect Structures in Co-Existence with the Ordered 2D Network.

All defect structures in co-existence with the ordered 2D network, such as non-equilateral hexagons, parallelograms, triangles and so on, consist of six types of structural units (Supplementary Figure 8a). The structural units include three types of "nodes": N-1 (Supplementary Figure 8b), N-2 (Supplementary Figure 8c) and N-3 (Supplementary Figure 8d), as well as three types of "edges": E-1 (Supplementary Figure 8e), E-2 (Supplementary Figure 8f) and E-3 (Supplementary Figure 8g).

Model for the 2D Structure with All Debromination Sites Involved in the PSP Nodes.

Supplementary Figure 9 presents the model of the 2D structure where all Br-substituted sites activated in the second step are involved in the PSP nodes. In this model, the ratio of the Ag atoms located at the hollow sites (light blue dots) to those at the bridge sites (dark blue dots) on Ag(111) is 1:2. This ratio in the hexagonal network becomes 2:1, as observed by STM. The occupancy of the hollow Ag atoms which are more stable in energy in the proposed structure is 33%, much lower than that (66%) in the experimentally observed 2D network, making the former less favorable in energy than the latter.

SUPPLEMANTRY REFERENCES

1. Hohenberg, P. & Kohn, W. Inhomogeneous electron gas. *Phys. Rev.* **136**, B864 (1964).
2. Kohn, W. & Sham, L. J. Self-consistent equations including exchange and correlation effects. *Phys. Rev.* **140**, A1133 (1965).
3. Frisch, M. J. et al. Gaussian 09. Revision A.02, Gaussian, Inc. (2009).
4. Becke, A. D. Density-functional thermochemistry. III. The role of exact exchange. *J. Chem. Phys.* **98**, 5648-5652 (1993).
5. Stephens, P. J., Devlin, F. J., Chabalowski, C. F. & Frisch, M. J. *Ab initio* calculation of vibrational absorption and circular dichroism spectra using density functional force fields. *J. Phys. Chem.* **98**, 11623-11627 (1994).
6. Dunning, T. H. & Hay, P.J. *Modern Theoretical Chemistry*, H. F. Schaefer III Ed. (Plenum Press, NY, 1977).
7. Hay, P. J. & Wadt, W. R. *Ab initio* effective core potentials for molecular calculations. Potentials for the transition metal atoms Sc to Hg. *J. Chem. Phys.* **82**, 270 (1985).
8. Wadt, W. R. & Hay, P. J. *Ab initio* effective core potentials for molecular calculations. Potentials for main group elements Na to Bi. *J. Chem. Phys.* **82**, 284 (1985).
9. Hay, P. J. & Wadt, W. R. *Ab initio* effective core potentials for molecular calculations. Potentials for K to Au including the outermost core orbitals. *J. Chem. Phys.* **82**, 299 (1985).
